# SuSPect: Enhanced Prediction of Single Amino Acid Variant (SAV) Phenotype Using Network Features

**DOI:** 10.1016/j.jmb.2014.04.026

**Published:** 2014-07-15

**Authors:** Christopher M. Yates, Ioannis Filippis, Lawrence A. Kelley, Michael J.E. Sternberg

**Affiliations:** Centre for Integrative Systems Biology and Bioinformatics, Imperial College London, London SW7 2AZ, UK

**Keywords:** MCC, Matthews correlation coefficient, SVM, support vector machine, PPI, protein–protein interaction, SAV, single amino acid variant, MSA, multiple sequence alignment, PSSM, position-specific scoring matrix, RSA, relative solvent accessibility, RBF, radial basis function, protein–protein interaction, nsSNP, missense mutation, SuSPect, SAV

## Abstract

Whole-genome and exome sequencing studies reveal many genetic variants between individuals, some of which are linked to disease. Many of these variants lead to single amino acid variants (SAVs), and accurate prediction of their phenotypic impact is important. Incorporating sequence conservation and network-level features, we have developed a method, SuSPect (Disease-*Su*sceptibility-based *S*AV Phenotype *P*r*e*di*ct*ion), for predicting how likely SAVs are to be associated with disease. SuSPect performs significantly better than other available batch methods on the VariBench benchmarking dataset, with a balanced accuracy of 82%. SuSPect is available at www.sbg.bio.ic.ac.uk/suspect. The Web site has been implemented in Perl and SQLite and is compatible with modern browsers. An SQLite database of possible missense variants in the human proteome is available to download at www.sbg.bio.ic.ac.uk/suspect/download.html.

## Highlights

• Bioinformatics approaches are key for identification of disease-causing variants.
• SAV phenotype prediction can be improved using network information.
• A method including these features, SuSPect, outperforms tested methods.
• SuSPect is available to use at www.sbg.bio.ic.ac.uk/suspect.

## Graphical abstract

**Figure:**
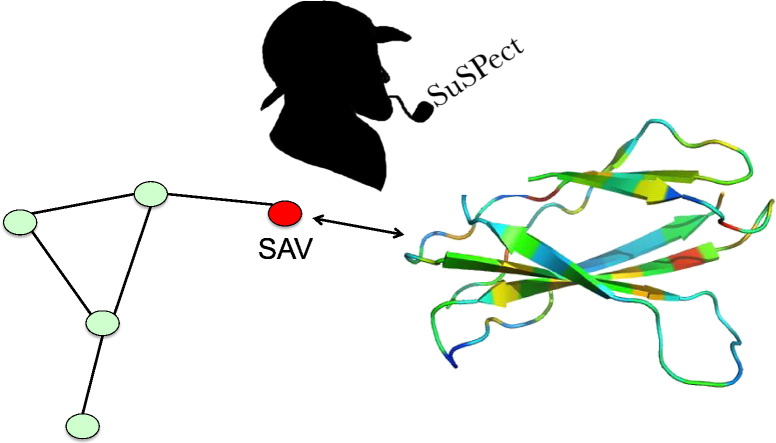

## Introduction

Large-scale projects, such as the Exome Sequencing Project† and the 1000 Genomes Project [1], have uncovered substantial genetic variation between individuals. Genome-wide association studies, whole-genome sequencing and exome sequencing have also been used to identify variants associated with both Mendelian diseases, such as cystic fibrosis, and complex diseases, including diabetes and cancer [2,3].

Non-synonymous single nucleotide variants are one of the best-studied groups of variants in human disease. These are single-base changes that lead to a change in the amino acid sequence of the encoded protein, termed a single amino acid variant (SAV) or missense variant. SAVs can also be caused by multiple nucleotide substitutions. The amino acid change can affect, for example, protein stability, interactions and enzyme activity, thereby leading to disease.

In a genome-wide association study or sequencing study, a large number of SAVs can be identified as potentially causative of a disease. It is not feasible to experimentally determine the phenotype and biochemical impact of such a large number of mutations; thus, accurate computational predictions are vital for analysis of identified SAVs. These predictions are generally based on sequence conservation, protein structural features or a combination of these, although other features have been included, such as Gene Ontology terms [4–9]. Commonly, these features are combined using machine learning methods such as random forests and support vector machines (SVMs).

We have previously shown that certain proteins and domains are significantly more likely than others to contain disease-associated variants [10]. *Disease-propensity* is based on a binomial test comparing the observed numbers of disease-associated and neutral variants in the protein/domain to random expectation. Predicting the phenotypic effect of variants based on the *disease-propensity* of the domain in which they are located can give good performance but may be affected by underlying biases in the training data toward well-studied proteins and the method has limited coverage of the human proteome. We showed that the susceptibility of proteins and domains to contain disease-associated variants is related to other features including the location in the interactome network of the protein or domain and the function of the protein. Thus, in this work, we include features that correlate with *disease-propensity*, such as protein–protein interaction (PPI) network centrality. To our knowledge, this is the first time PPI network-based features have been used for SAV phenotype prediction.

The function of a folded protein is intimately linked to its three-dimensional structure, and in many cases, the effects of SAVs can be understood by investigating their effects on the protein structure. Accordingly, several approaches to predict phenotype include structural features, particularly when evolutionary information is lacking [6,11,12]. For example, many disease-associated SAVs are located in the core of the protein, whereas SAVs on the surface are more likely to be neutral [13]. However, variants on the surface can affect PPIs, leading to disease [14,15]. In a recent review, we summarized the various mechanisms by which SAVs can affect PPIs, including steric clashes, loss of salt bridges, changes in intrinsic disorder and alterations in post-translational modifications [16].

There is currently low structural coverage of the human proteome and consequently structure-based prediction is only possible for a subset of variants. To counter this, we use both experimentally solved protein structures and homology models produced by Phyre2 [17], greatly increasing structural coverage of the proteome. Structural features may be useful in assessing the likely impact of an SAV, and in this work, we also test whether or not structural information can add value to SAV phenotype prediction.

In our approach, we combine sequence and structural features, which are used in other widely used algorithms, with several other features including network information to train an SVM to identify disease-associated SAVs. Because many of the features chosen are related to the differences between *disease-susceptible* and *disease-resistant* domains and proteins, we have termed our method SuSPect (Disease-*Su*sceptibility-based *S*AV Phenotype *P*r*e*di*ct*ion).

We find that incorporating PPI network information improves predictive performance. Surprisingly, features derived from the three-dimensional structure of a protein do not contribute to performance. This is not due to the low structural coverage of the proteome, as the same pattern is seen when tested only on those SAVs with an experimentally solved structure available. Protein structures can, however, help with human interpretation.

SuSPect has been trained to determine the likelihood of an SAV to be associated with disease, and when tested on the VariBench benchmarking dataset [18], SuSPect shows greatly improved performance compared to other widely used methods allowing batch submission, such as PolyPhen-2 [8], SIFT [19], MutationAssessor [20], Condel [9] and FATHMM [21]. Feature selection is used to further improve performance. SuSPect is available as a Web server, where users can submit individual mutations or a VCF (*v*ariant *c*all *f*ormat) file or download a database of pre-calculated scores for all possible SAVs in the human proteome.

## Results

A total of 77 features (see Table S1) were calculated for 20,728 disease and 36,799 polymorphism SAVs from the Humsavar database and these SAVs were used to train the SVM learning algorithm, SuSPect-All. Table 1 shows the number of SAVs that could be mapped to PDB structures and Phyre2 models. Using these homology models, we have been able to increase structural coverage from 7.6% to 60.4% of SAVs, an 8-fold increase. A structure or model is available for 48% of the human proteome.

SAVs that could be mapped to PDB structures are significantly more likely to be annotated as disease-associated than those in Phyre2 models or with no structure available (χ^2^ test, *p* < 2.2 × 10^− 16^), while those for which a Phyre2 model is available are more likely to be disease-associated than those with no structural information available (χ^2^ test, *p* < 2.2 × 10^− 16^). These results may be due to an intrinsic bias in either the PDB database or the SAV database, with disease-associated proteins more likely to have been studied in detail and therefore to have an available structure or the structure of a homologue.

### Feature selection

Stability selection [22] was used in conjunction with mRMR (*m*inimum *r*edundancy, *m*aximum *r*elevance) [23] to select the most important features. In 10-fold cross-validation, performance following feature selection was similar to that using all features according to Matthews correlation coefficient (MCC) and balanced accuracy (Table S2). Feature selection was carried out on the full training set, with nine features chosen in all stability selection subsets. These features are described in Table 2 and include a combination of sequence conservation, predicted solvent accessibility and protein network centrality. These features were used to train an SVM, hereafter termed SuSPect-FS (for feature selection). As shown below, SuSPect-FS outperforms SuSPect-All on unseen data. Carrying out feature selection separately on the three sets of SAVs (grouped according to the availability of a structure) gives worse performance than when carried out on all SAVs together (DeLong's test, *p* < 2.2 × 10^− 16^).

Degree centrality in a PPI network is selected as an important feature. We have previously shown that proteins with significantly more disease-associated than neutral SAVs (*disease-susceptible*) are positioned centrally in PPI networks [10]. SAVs can affect protein function without leading to disease, for example, if normal cellular function can be carried out even in the complete absence of the protein [24]. We and others have found that mutations in more centrally positioned proteins are more likely to be associated with disease; thus, PPI centrality is likely to be important in discriminating between mutations affecting proteins unlikely to be involved in disease from those in proteins whose mutation is likely to lead to disease. The importance of PPI centrality is shown by the fact that all four centrality measures used are ranked in the top 25% of features (Table S1). There may be bias toward well-studied proteins, although the PPI network used was filtered to only contain those interactions with experimental evidence, which should lessen any bias.

Sequence conservation and predicted solvent accessibility have previously been shown to be useful in SAV phenotype prediction [5]. Functional annotations from UniProt are used to identify variants affecting functionally important residues, for example, those that bind to metal ions or are in a disulfide bond. Because these residues have important functional roles, their mutation can lead to impaired function and therefore disease. While better-studied proteins are potentially more likely to be annotated, many annotations in the UniProt FT table come from similarity or predictions rather from direct observations, which should decrease the bias toward better-studied proteins. Jensen-Shannon divergence is an information-theoretic measure for identifying important residues by comparing the observed distribution of amino acids in a multiple sequence alignment (MSA) with an estimated background distribution. Positions differing from this background are assumed to be under evolutionary pressure, constraining the observed distribution of amino acids [25].

### Performance of SuSPect

**Fig. 1:**
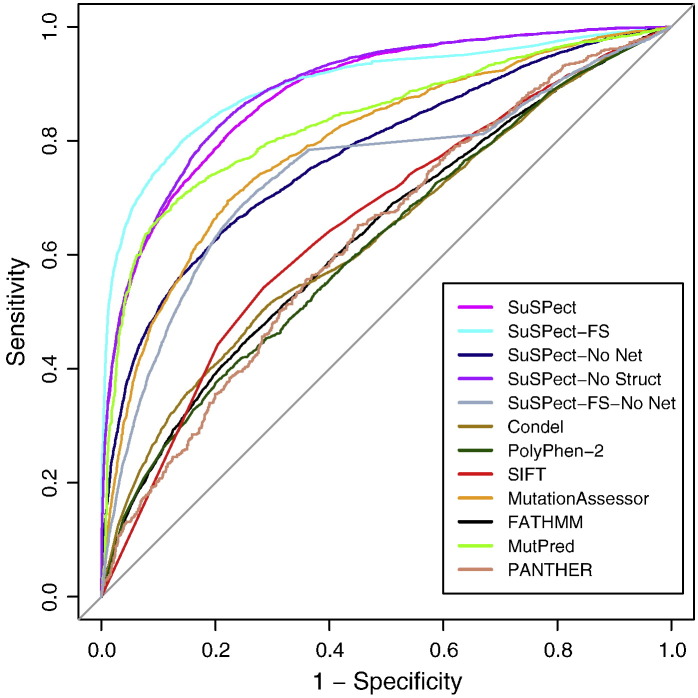
Performance of five versions of SuSPect compared to seven other methods.

As a test on previously unseen data, the neutral and pathogenic datasets were downloaded from VariBench and filtered to remove any SAVs present in the SuSPect training set, leaving 5432 pathogenic and 13,236 neutral SAVs. This dataset was chosen because of its size and because it has previously been used for benchmarking of similar methods [26]. Figure 1a shows ROC (*r*eceiver *o*perating *c*haracteristic) curves comparing the performances of SuSPect-All and SuSPect-FS with those of FATHMM, PolyPhen-2, SIFT, Condel, MutationAssessor, MutPred and PANTHER with AUC (*a*rea *u*nder *c*urve), balanced accuracy, precision, recall and MCC shown in Table 3. The first five of these methods were chosen based on the availability of a Web server allowing batch submission, reflecting the situation faced by a user with a large number of SAVs to analyze and filter. Scores for MutPred and PANTHER were obtained from Thusberg *et al.* [26]. Other methods, such as FunSAV [27], have shown good performance in benchmarking but are unavailable for batch submission or are only applicable to a subset of SAVs, such as those with an experimentally solved structure available.

In this benchmark, SuSPect-All and SuSPect-FS outperform other tested methods, achieving higher sensitivity without loss of selectivity. High sensitivity corresponds to a high proportion of disease-associated SAVs being correctly classified, which SuSPect is able to do without increasing the number of false positives. Feature selection improves performance: SuSPect-FS has an AUC of 0.90, which is significantly higher than SuSPect-All (Fig. 1a, DeLong's test [28], *p* < 10^− 10^). In addition to these methods, SNAP, SNPs&GO, PHD-SNP and SNPanalyzer results were obtained from Thusberg *et al.* [26]. In these cases, results were provided as binary classifications, meaning ROC curves could not be produced, but other performance measures (precision, recall, balanced accuracy and MCC) are shown in Table 3, together with the methods mentioned previously.

SuSPect-FS has the highest AUC and MCC, as well as the joint-highest balanced accuracy. These three are balanced measures of performance and thus are unaffected by the discrepancy in number of neutral and pathogenic SAVs in the dataset. SNPs&GO shows the highest precision and *F*-measure, meaning a user can be confident that if an SAV is predicted to be associated with disease, that is likely to be the case. However, SNPs&GO shows lower recall than SuSPect-FS; thus, more disease-associated SAVs are incorrectly classified as neutral, as well as a lower MCC, which is a measure of how much of the data overall falls in the true negative and true positive categories. A higher value therefore corresponds to higher confidence that SAVs called as disease-associated or neutral are truly disease-associated or neutral, respectively. While three methods (SNAP, SNPs&GO and MutPred) show higher *F*-measure than SuSPect-FS, it is worth noting that this measure does not take into account the true negative rate of predictions, unlike MCC, which is therefore a better measure of overall performance, taking into account how often neutral variants are correctly classified as such.

As a test of the importance of network centrality, we removed all network-related features (network centralities and protein–protein interface information) from SuSPect-All and retrained without these features. This SVM gives significantly worse performance than the full method (SuSPect-No Network, DeLong's test, *p* < 2.2 × 10^− 16^). Performance without network features is inferior to that of MutationAssessor (AUC, DeLong's test, *p* < 0.01), MutPred (AUC, *p* < 10^− 13^) and SNPs&GO, showing that it is the use of network features that improves performance over other methods. Similar results are seen upon removing PPI network centrality from SuSPect-FS (its only network-related feature), with an AUC of 0.74 (*p* < 2.2 × 10^− 16^), MCC of 0.38 and balanced accuracy of 0.67, all considerably lower than when PPI network centrality is included.

Interestingly, no structural features were selected for inclusion in SuSPect-FS, suggesting that these features do not give any extra information over that provided by the sequence. To assess this, we removed all structural features from SuSPect-All and retrained the SVM. On the VariBench dataset, this gives slightly better performance than SuSPect-All (DeLong's test, *p* < 10^− 14^) although worse than SuSPect-FS (SuSPect-No Structure, DeLong's test, *p* < 10^− 3^; Fig. 1 and Table 3). In cross-validation, performance is similar to that of SuSPect (Table S3). The lack of importance of structural features is not due to the poor structural coverage; an SVM was trained only on those SAVs that have a structure available from the PDB. This SVM performs the same as SuSPect-All on the VariBench SAVs with a PDB structure available (Supplementary Table S4, DeLong's test, *p* = 0.60). Interestingly, when only predicted solvent accessibility is included, performance is slightly but statistically significantly better than when the NACCESS calculated solvent accessibility is included (AUC = 0.91 and 0.90, respectively; DeLong's test, *p* < 10^− 3^). One possible explanation for this is that NetSurfP may be providing information about protein quaternary structure, whereas the NACCESS solvent accessibility calculated from a monomeric structure will not. While structural features may not aid predictive performance, they are helpful in interpretation of how a mutation may have its effect, thus are included in the output of the SuSPect Web server (see below).

### Web server and download

**Fig. 2:**
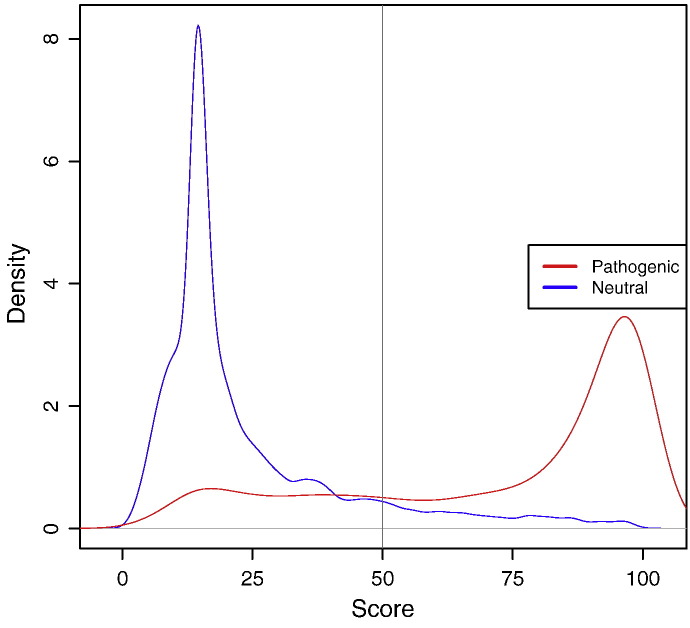
Distribution of SuSPect-FS scores for disease-associated (red) and neutral (blue) SAVs in the VariBench test set. The two sets of SAVs have significantly different distributions (Wilcoxon test, *p* < 2.2 × 10^− 16^).

SuSPect is available‡ for non-commercial use. Pre-calculated scores from SuSPect-FS are available for human mutations, queried using UniProt accessions or by uploading a VCF file. Scores range from 0 to 100, with a recommended cutoff of 50 for discriminating between neutral and disease-associated SAVs. The distribution of SuSPect scores on the VariBench test set is shown in Fig. 2. In addition to giving a score, detailed information about the SAV is provided, including an image of the SAV in a protein structure or model where available. This extra information, including predicted post-translational modifications, Pfam domains and sequence conservation, helps interpretation of the scores, which we see as being particularly helpful in determining which SAVs are functionally important in disease. These pre-calculated scores are also available to download as an SQLite database to allow users to obtain scores for SAVs locally without uploading large files or potentially sensitive information. If an SAV of known phenotype is uploaded, we inform the user of the phenotype using information from databases such as OMIM or dbSNP.

Alternatively, users can upload a sequence or structure and receive scores for all possible mutations at all positions. The Humsavar database used for training data consists mostly of nsSNPs (*n*on-*s*ynonymous *s*ingle *n*ucleotide *p*olymorphisms), which are SAVs brought about by only a single-base change, although there are 164 examples of SAVs requiring multiple base substitutions. Because there may be differences between nsSNPs and other SAVs and SuSPect has been trained primarily on nsSNPs, we highlight SAVs that cannot be reached by an nsSNP as part of the extra detailed information. Scores are obtained from the SuSPect-FS database for human proteins and using SuSPect-No Networks for proteins from other organisms, which lack PPI network centrality information. Where a structure has been provided, this can be viewed interactively using JSmol [29], with user-selected residues of interest highlighted.

Scores range from 0 (neutral) to 100 (disease-associated), with more extreme scores corresponding to a greater degree of confidence in the prediction. The distributions of scores in the VariBench test set are shown in Fig. 2.

## Discussion

We have developed SuSPect, a new method for predicting whether an SAV is associated with disease. Sequence conservation and solvent accessibility are known to be important determinants of the likelihood for an SAV to be deleterious [13,30]. In addition, we have previously shown that *disease-susceptible* proteins, in which SAVs are significantly more likely to be disease-associated than expected by chance, are located more centrally in PPI networks according to betweenness, degree and coreness centralities [10]. As such, network centrality helps to discriminate between disease-associated and tolerated SAVs by describing how likely any variation in the protein is to lead to disease. Removing network-based features gives a large drop in performance, showing that these features are important for our improved prediction of phenotype. Another important difference is that SuSPect has been specifically trained to discriminate between disease-associated and neutral SAVs, as opposed to predicting an effect on protein function. This is because genetic variants can affect protein function without leading to disease [24] and, while loss of function is often used as a proxy for disease, it is better to use a tool specifically designed for the task. In spite of this, SuSPect is still able to outperform SIFT at predicting the phenotypic effects of a set of mutations in non-human proteins (Supplementary Table S5).

Most of the SAVs used to train SuSPect are involved in Mendelian diseases, although there are some variants involved in complex disorders. While this may lead to difficulty of interpretation when used on complex diseases, many of the same principles could apply between Mendelian and complex phenotypes, and the SuSPect score could be a useful way of prioritizing variants for further investigation.

Using Phyre2 structural models, we increased the structural coverage of the human proteome. Only 7.6% of SAVs in our training data could be mapped to a structure from the PDB, but by also using Phyre2 models, 60.4% of variants had a structure available. However, we see no significant increase in performance when structural features are included and no structural features are chosen through feature selection, suggesting that the sequence of a protein contains sufficient information about protein structure for SAV phenotype prediction. For human interpretation, however, the sequence signal is highly complex and interpretation is problematic; thus, structural information can be helpful.

On a blind test, SuSPect-All and SuSPect-FS significantly outperform PolyPhen-2, SIFT, MutationAssessor, Condel and FATHMM. SuSPect-FS has an AUC of 0.90, which is significantly higher than all other methods tested. We have also tested its ability to predict the phenotypes of mutations in non-human proteins and seen good performance, although worse than on human proteins due to the lack of PPI network information and the fact that predicting a loss of protein function is not the same as predicting a disease-associated mutation, which is the task for which SuSPect was developed. A further test of SuSPect would be the CAGI (*C*ritical *A*ssessment of *G*enome *I*nterpretation§) experiment. A previous, development-stage version of SuSPect was entered into the CAGI 2012 experiment; thus, we would hope to see improved performance in future assessments.

An example of an SAV showing the potential importance of network centrality for phenotype prediction is p.Cys873Gly in MSH2 (UniProt: P43246), which has been identified in families with gastric cancer [31]. This position is not highly conserved, with low Jensen-Shannon entropy and only a small decrease in position-specific scoring matrix (PSSM) score (from 2 to − 1), and is not predicted to be buried. However, MSH2 has high degree in the STRING PPI network, interacting with a number of cancer-related proteins, such as PCNA and MLH1. Because of this high degree, SuSPect-FS predicts this SAV to be deleterious (score = 69), whereas SIFT (0.52), PolyPhen-2 (0.003), Condel (0.002), MutationAssessor (1.87), PANTHER (0.43624) and MutPred (0.395) all predict that it will be tolerated. While this is only a single example, it does suggest that there are cases where SuSPect can identify deleterious variants that would be missed by other methods. One potential limitation of using network centrality is the potential bias toward well-studied proteins, although using data from high-throughput experiments should lessen this bias.

Unlike other methods, the SuSPect Web server also provides users with an explanation of the features and annotations associated with the SAV, which can aid understanding of why a mutation is predicted to be deleterious or not. If the SAV is present in the training data, this will also be noted and the phenotype returned. By providing improved SAV phenotype prediction performance compared to other methods, we consider SuSPect will be a useful tool for research into disease, protein evolution and protein structure.

## Materials and Methods

### SAV data

SAVs were downloaded from Humsavar|| (version 2011–09) and VariBench [18]. In the Humsavar database from UniProt-KB, mutations are annotated as Disease, Polymorphism or Unclassified, depending on whether they are disease-associated, neutral or of unknown phenotype. The VariBench neutral dataset is from dbSNP and the pathogenic dataset is from PhenCode, which collates mutations from SwissProt and numerous locus-specific databases [32]. Where necessary, VariBench SAVs were mapped to UniProt sequences based on mapping between UniProt, GenBank and RefSeq accessions and using BLASTP to align sequences. For the blind test, SAVs also present in the Humsavar database were removed, leaving 5432 pathogenic and 13,236 neutral SAVs, many of which are in proteins also present in the training data. However, in cross-validation, we did not see over-training due to protein-level features (see Supplementary Table S2).

Condel [9], PolyPhen-2 [8], SIFT [19] and MutationAssessor [20] scores were obtained from the Condel Web server¶ and FATHMM [21] scores were from the FATHMM Web server^a^. PolyPhen-2 provides both scores and a classification, but we only use the scores in our analysis. These methods were chosen based on the availability of batch submission. Predictions from MutPred, PANTHER, PHD-SNP, SNAP, SNPanalyzer and SNPs&GO were obtained from Thusberg *et al.* [26].

### Protein structures and models

To obtain protein structures, we used the mapping file pdb2sp.txt from UniProt. PDB files were filtered to remove those containing multiple chains, meaning only monomeric structures were used, preventing any misinterpretation of a position as buried when it is in fact at an interface. For each SAV, the UniProt sequence was aligned to the PDB sequence using BLASTP [33]. We required the wild-type amino acid to match the amino acid in the PDB file. If multiple structures were available, that with the best resolution was used.

Where PDB structures were not available, Phyre2 structural models from the Genome3D project were used, requiring a confidence of at least 90% in the model [17,34]. Phyre2 uses HMM-HMM (*h*idden *M*arkov *m*odel) alignments to compare a protein sequence to proteins in a fold library. If a match can be found, the query structure is modeled on the matching structure. If no model could be generated covering an SAV position, only sequence-based features were used for prediction.

### SVM features

We used a total of 77 features in SuSPect-All, which were then reduced to nine by feature selection (see section 2.5). Previous studies have shown the importance of sequence conservation in SAV phenotype prediction. To this end, we obtained PSSMs and MSAs by running PSI-BLAST and storing the PSSM produced after three iterations and the MSA after a single iteration [35]. PSSMs are substitution matrices showing, for each position in the protein, how likely each amino acid is to occur, based on their frequencies in an alignment. Uniref50 was used as the sequence database as it has been suggested that it can improve performance in homology detection [36]. The best (measured by lowest *E*-value) sequences to (i) have any amino acid other than the wild type or (ii) have the mutant amino acid at an SAV position were found. Their BLAST *E*-values and sequence identities to the query sequence were used as features. These features show how far away two protein sequences have diverged in total before the SAV position changes and the new amino acid is observed. The MSA was also used to calculate Jensen-Shannon divergence for all columns with fewer than 99.9% gaps, and the proportion of gaps in a column was used as another feature [25]. Multiple sequence conservation-based features are used because they each provide differing information. For example, Jensen-Shannon divergence is a measure of conservation at a specific position of an MSA, whereas the sequence identity-based features show how far two protein sequences have diverged overall in order for a given variant to occur between them.

Structural features are also thought to be useful in SAV phenotype prediction. Where possible, SAVs were mapped to monomeric PDB structures or homology models produced using Phyre2. For each structure, DSSP was used to give secondary structure, ϕ/ψ backbone torsion angles and backbone hydrogen bonds [37] and NACCESS^b^ was used for relative solvent accessibility (RSA). Fpocket was used to find surface pockets [38]. Betweenness centrality on a residue interaction network was calculated using the igraph library for R [6,39]. Residue interaction networks were produced by connecting all amino acids located < 5 Å from one another. Catalytic sites from the Catalytic Site Atlas [40] and protein interface residues from PISite [41] and ProtInDB^c^ were obtained for PDB structures. For Phyre2 models, these annotations were mapped from the template structure.

NetSurfP was used to predict secondary structure and RSA based on the results of a PSI-BLAST search [42]. IUPred was used to identify disordered regions [43] and ANCHOR was used to identify disordered binding sites in both the wild type and mutant sequences [44]. Aliphatic index and GRAVY (*gr*and *av*erage of h*y*dropathy) were calculated for the wild-type sequence as described in Refs. [45] and [46], respectively. The former may be related to protein stability, while GRAVY characterizes the hydrophobicity of a protein.

SAVs located in protein domains are more likely to be deleterious than those elsewhere (e.g., in linkers), and the change in Pfam *E*-value caused by the SAV has been used previously to predict its effects [47]. Domains were detected using Pfam and, if a residue was in a domain, the emission probability for the wild type and the mutant amino acid at that position of the Pfam HMM was obtained [48].

The centrality of each domain in both a domain–domain interaction (from DOMINE [49]) and a domain bigram network [50] were calculated using four measures: betweenness, closeness, coreness and degree. The same four centrality measures were calculated for each protein in a PPI network obtained from STRING by filtering for human interactions with experimental evidence [51]. In a network, the shortest path between two nodes (protein or domain) is the path that requires the fewest edges. Betweenness centrality is the proportion of all shortest paths passing through a given node. Closeness centrality is the inverse of the sum of all shortest paths, showing how far away the node is from all other nodes. Coreness is defined using *k*-core centrality [52], an iterative process in which nodes and their adjacent edges are removed from the network if their degree is less than an integer *k*. This is repeated until all remaining nodes have degree of at least *k*, with these nodes constituting the *k*-core. The coreness of a node is the highest value of *k* for which it is present in the *k*-core but not the *k* + *1*-core. Degree centrality is the number of edges adjacent to a node (e.g., the number of interactions a protein makes).

Functional annotations were obtained from the UniProt FT table [53]. Finally, a number of features describing the wild type and the mutant amino acids were used—BLOSUM [54], change in charge, mutation to/from glycine/proline and changes to the values of the five principle components calculated by Atchley *et al.* [55] from the 494 amino acid indices in the Amino Acid Index [56].

LIBSVM 3.12 was used for SVMs, using a radial basis function (RBF) kernel [57]. RBF kernel SVMs have two parameters—C, which is the soft margin parameter, determining how much misclassifications in the training set are punished, and gamma, which is the radius of the RBF kernel, determining how far the influence of each training data point stretches. The *sigest* function in the *kernlab* library was used to obtain a suitable value for gamma [58], and 10-fold cross-validation was used to determine the optimum value for C. Following cross-validation, C = 128 and gamma = 0.01 were chosen.

While there are protein-level features present, when cross-validation was carried out by dividing proteins into 10 sets, there was only a very small drop in MCC and balanced accuracy compared to dividing the SAVs into 10 groups with proteins present in multiple groups (see Supplementary Table S2), implying that there is little or no over-training for certain proteins.

A model was trained using the SAVs from Humsavar and tested on those from VariBench, after filtering to remove any SAVs also present in the training set. Probability estimates were obtained by using the − b 1 option when training the SVM, as described in Ref. [57]. These probability estimates were then multiplied by 100 to give the SuSPect score. Before training and testing, all features were scaled to between 0 and 1.

### Evaluation of performance

We carried out 10-fold cross validation as an initial test (see Supplementary Information). As a test on unseen data, VariBench was used, having been filtered to remove SAVs present in the training data. ROC curves and their AUC were calculated using the *pROC* package in R [59]. ROC curves were compared using the *roc.test* function, which implements DeLong's non-parametric test for AUC comparison [28]. Accuracy, recall (selectivity), precision, MCC, *F*-measure and balanced accuracy were calculated using the following equations, where *TP* is the number of correctly predicted disease SAVs, *TN* is the number of correctly predicted neutral polymorphisms, *FP* is the number of neutral SAVs misclassified as disease and *FN* is the number of disease SAVs classified as neutral. For cross-validation, the SVM was trained to give a binary classification. For blind testing, probabilistic outputs were used, with a cutoff of 50 used to discriminate between neutral and disease-associated SAVs.

$$\begin{array}{l} \begin{array}{ccc} \mathrm{Accuracy}=\frac{\mathrm{TP}+\mathrm{TN}}{\mathrm{TP}+\mathrm{TN}+\mathrm{FP}+\mathrm{FN}} & \mathrm{Recall}=\frac{\mathrm{TP}}{\mathrm{TP}+\mathrm{FN}} & \mathrm{Precision}=\frac{\mathrm{TP}}{\mathrm{TP}+\mathrm{FP}} \end{array}\\ \begin{array}{cc} \mathrm{Balanced}\;\mathrm{Accuracy}=\frac{0.5\times \mathrm{TP}}{\mathrm{TP}+\mathrm{FN}}+\frac{0.5\times \mathrm{TN}}{\mathrm{TN}+\mathrm{FP}} & \mathrm{MCC}=\frac{\mathrm{TP}\times \mathrm{TN}-\mathrm{FP}\times \mathrm{FN}}{\sqrt{\left(\mathrm{TP}+\mathrm{FP}\right)\left(\mathrm{TP}+\mathrm{FN}\right)\left(\mathrm{TN}+\mathrm{FP}\right)\left(\mathrm{TN}+\mathrm{FN}\right)}} \end{array}\\ F‐\mathrm{measure}=\frac{2\times \mathrm{Precision}\times \mathrm{Recall}}{\mathrm{Precision}+\mathrm{Recall}} \end{array}$$

### Feature selection

For feature selection, stability selection was used in conjunction with mRMR. In stability selection, feature selection is carried out on multiple subsets of 50% of the SAVs and the frequency at which each feature is chosen is used to determine important features [22]. mRMR aims to identify the subset of features most relevant to the outcome while reducing the redundancy between selected features [23]. In 10-fold cross-validation, SAVs were split into 10 sets. Feature selection was carried out on nine of the sets and then an SVM trained on these sets with the selected features. This SVM was used to make predictions on the tenth set. This was repeated for all test sets. In both cross-validation and feature selection on the full training set, 100 subsets were used for stability selection, with those features selected in all 100 iterations chosen. For mRMR, parameter alpha was set to 0.5 and 30 features were selected in each iteration.

Unless otherwise stated, statistical tests and calculations were carried out using R and scripting with Perl.

## Figures and Tables

**Table 1:** Distribution of disease and polymorphism SAVs in PDB and Phyre2 structures

Phenotype	Structure			Total
	PDB	Phyre2	N/A	
Disease	2914	13,560	4254	20,728
Polymorphism	1468	16,833	18,498	36,799
Total	4382	30,393	22,752	57,527

**Table 2:** Features chosen in feature selection on the full training set

Feature
(a) Degree centrality in a PPI network.
(b) Number of annotations at this position in UniProt FT feature table.
(c) Score for the wild-type amino acid in a PSSM.
(d) Score for the mutant amino acid in PSSM.
(e) Difference between PSSM scores for the wild type and mutant amino acids at the SAV position.
(f) Difference between Pfam HMM emission probabilities for the wild type and mutant amino acids at the SAV position.
(g) Jensen-Shannon divergence, a measure of sequence conservation.
(h) Percentage sequence identity with the first sequence in the MSA to have the mutant amino acid at the SAV position.
(i) RSA predicted by NetSurfP.

**Table 3:** Performance of five versions of SuSPect compared to 11 other SAV phenotype prediction methods, ordered by MCC

Method	Precision	Recall	*F*-measure	Balanced accuracy	MCC	AUC
SuSPect-FS	0.75	0.75	0.75	**0.82**	**0.65**	**0.90**
SuSPect-No Structure	0.73	0.67	0.70	0.79	0.59	0.89
SuSPect-All	0.72	0.67	0.69	0.78	0.58	0.88
SNPs&GO	**0.96**	0.70	**0.81**	**0.82**	0.56	—
MutPred	0.79	**0.81**	0.80	0.75	0.49	0.84
SuSPect-No Networks	0.78	0.64	0.70	0.71	0.44	0.78
PHD-SNP	0.69	0.72	0.70	0.69	0.39	—
MutationAssessor	0.36	**0.81**	0.50	0.70	0.34	0.79
SuSPect-FS-No Networks	0.63	0.45	0.53	0.67	0.38	0.74
SNAP	0.82	0.75	0.78	0.68	0.34	—
FATHMM	0.41	0.71	0.52	0.63	0.24	0.63
SIFT	0.14	0.58	0.23	0.62	0.22	0.65
Condel	0.43	0.52	0.47	0.61	0.21	0.63
SNPanalyzer	0.94	0.61	0.74	0.65	0.20	—
PANTHER	0.43	0.75	0.55	0.59	0.17	0.63
PolyPhen-2	0.37	0.60	0.46	0.58	0.14	0.62

For four methods, predictions were binary; thus, AUC could not be calculated.

## Footnotes

† http://evs.gs.washington.edu/EVS/

‡ at www.sbg.bio.ic.ac.uk/suspect

§ http://www.genomeinterpretation.org

|| http://www.uniprot.org/docs/humsavar.txt

¶ http://bg.upf.edu/condel/analysis

a http://fathmm.biocompute.org.uk/inherited.html

b http://www.bioinf.manchester.ac.uk/naccess/

c http://protindb.cs.iastate.edu/index.py
