# Supplementary material for: SuSPect: Enhanced Prediction of Single Amino Acid Variant (SAV) Phenotype Using Network Features
Source: J Mol Biol. 2014 Jul 15;426(14):2692–701. doi: 10.1016/j.jmb.2014.04.026 (PMC4087249; doi:10.1016/j.jmb.2014.04.026)
Supplement: Supplementary file 1 — Supplementary material. [file mmc1.docx]

| **Feature** | **Frequency Selected** | **Ranking** | **Range** |
| --- | --- | --- | --- |
| Position-specific scoring matrix (PSSM) score for wild-type | 100 | =1 | -8-14 |
| PSSM score for mutant | 100 | =1 | -13-13 |
| Change in PSSM score | 100 | =1 | -19-26 |
| Protein-protein interaction (PPI) network degree centrality (Szklarczyk *et al.,* 2011) | 100 | =1 | 0-13,610 |
| Sequence ID with first sequence to have mutant amino acid | 100 | =1 | 0-99.86 |
| UniProt FT features (The UniProt Consortium, 2013) | 100 | =1 | 0-5 |
| Change in Pfam Hidden Markov model (HMM) score (Punta *et al.*, 2012) | 100 | =1 | -12.2-9.3 |
| Jensen-Shannon divergence (Capra & Singh, 2007) | 100 | =1 | 0-0.90 |
| Predicted relative solvent accessibility (NetSurfP Petersen *et al.*, 2009)) | 100 | =1 | 0-0.95 |
| Sequence ID with first sequence to lack wild-type amino acid | 99 | =10 | 0-99.83 |
| BLOSUM62 score of amino acid change | 99 | =10 | -4-3 |
| PPI network coreness centrality | 96 | 12 | 0-568 |
| PPI network betweenness centrality | 93 | 13 | 0-2,320,311 |
| Catalytic Site Atlas (Porter *et al.*, 2004) | 89 | 14 | 0/1 |
| Information score for PSSM column | 87 | 15 | -0.04-5.7 |
| PPI network closeness centrality | 83 | 16 | 0-3.215x10^-5^ |
| Wild-type amino acid is Gly | 82 | 17 | 0/1 |
| Mutant amino acid is Pro | 80 | 18 | 0/1 |
| No structural information | 79 | 19 | 0/1 |
| BLAST E-value of first sequence to lack wild-type amino acid | 76 | 20 | 0-9.3 |
| Change in side-chain charge | 74 | 21 | -2-2 |
| Domain-domain interaction (DDI) network closeness centrality | 71 | 22 | 0-1.2x10^-7^ |
| IUPred score for wild-type sequence (Dosztányi *et al.*, 2005) | 70 | 23 | 0-1 |
| BLAST E-value of first sequence to have mutant amino acid | 68 | 24 | 0-9.9 |
| PDB structure | 67 | 25 | 0/1 |
| Crystallographic B factor | 59 | =26 | 0-279 |
| Pfam HMM score for mutant | 59 | =26 | 0-12.2 |
| ProtInDB protein interface | 55 | 28 | 0/1 |
| Surface pocket (fpocket (Le Guilloux *et al.*, 2009)) | 54 | 29 | 0/1 |
| GRAVY (grand average of hydropathy (Kyte & Doolittle, 1982)) | 44 | 30 | -1.3-1.6 |
| DDI network coreness centrality | 38 | 31 | 1-62 |
| Change in AAIndex principle component (PC) 1 (Atchley *et al.*, 2005) | 36 | =32 | -3.17-3.17 |
| Betweenness centrality in a residue interaction network | 36 | =32 | -1.4-14.5 |
| Change in ANCHOR score (Meszaros *et al.*, 2009) | 30 | 34 | -1-1 |
| Domain bigram network closeness centrality | 28 | 35 | 0-2.3x10^-7^ |
| Probability of beta strand (NetSurfP) | 26 | 36 | 0-0.98 |
| IUPred score for mutant sequence | 25 | =37 | 0-1 |
| UniProt TRANSMEM | 25 | =37 | 0/1 |
| Probability of alpha helix (NetSurfP) | 24 | =39 | 0-1 |
| Mutant amino acid is Gly | 24 | =39 | 0/1 |
| Secondary structure is turn (DSSP:ST (Kabsch & Sander, 1983)) | 22 | 41 | 0/1 |
| H-bond energy (N2) | 18 | 42 | -9.9-0 |
| H-bond energy (O2) | 16 | =43 | -3.5-0 |
| Secondary structure is strand (DSSP:BE) | 16 | =43 | 0/1 |
| Psi backbone angle | 16 | =43 | -180-360 |
| Domain bigram network betweenness centrality | 16 | =43 | 0-570,835 |
| H-bond energy (N1) | 14 | 47 | -9.9-0 |
| PISite protein interface (27) | 12 | 48 | 0/1 |
| H-bond energy (O1) | 11 | 49 | -9.9-0 |
| In a Pfam domain | 9 | 50 | 0/1 |
| Proportion of gaps in multiple sequence alignment column | 7 | =51 | 0-0.9992 |
| Aliphatic index (Ikai, 1980) | 7 | =51 | 7.5-152 |
| Domain bigram network degree centrality | 7 | =51 | 0-159 |
| Change in AAIndex PC 4 | 6 | 54 | -3.4-3.4 |
| Phi backbone angle | 5 | =55 | -180-360 |
| Wild-type amino acid is Pro | 5 | =55 | 0/1 |
| ANCHOR probability for wild-type sequence | 4 | =57 | 0-1 |
| ANCHOR probability for mutant sequence | 4 | =57 | 0-1 |
| ANCHOR score for wild-type sequence | 4 | =57 | 0/1 |
| ANCHOR score for mutant sequence | 4 | =57 | 0/1 |
| Change in AAIndex PC 2 | 4 | =57 | -3.4-3.4 |
| Change in AAIndex PC 3 | 4 | =57 | -7.9-7.9 |
| DDI network degree centrality | 4 | =57 | 0-499 |
| Change in AAIndex PC 5 | 3 | 64 | -6.1-6.1 |
| Secondary structure is helix (DSSP:GHI) | 2 | =65 | 0/1 |
| Probability of coil (NetSurfP) | 2 | =65 | 0-1 |
| DDI network betweenness centrality | 1 | =67 | 0-1,460,304 |
| Polar RSA (NACCESS, http://www.bioinf.manchester.ac.uk/naccess/) | 1 | =67 | 0-461 |
| Domain bigram network coreness centrality | 0 | =69 | 1-11 |
| Pfam HMM score for wild-type | 0 | =69 | 0-9.65 |
| Change in IUPred scores | 0 | =69 | -0.96-0.27 |
| Change in ANCHOR probability | 0 | =69 | -0.99-0.49 |
| All-atom RSA (NACCESS) | 0 | =69 | 0-188 |
| Side-chain RSA (NACCESS) | 0 | =69 | 0-181 |
| Main-chain RSA (NACCESS) | 0 | =69 | 0-631 |
| Non-Polar RSA (NACCESS) | 0 | =69 | 0-294 |
| Phyre2 model confidence (Kelley & Sternberg, 2009) | 0 | =69 | 90-100 |

**Table S1**

Features used in SuSPect-All, together with the number of times they are selected in stability selection (Meinshausen & Bühlmann, 2010) using mRMR (Ding & Peng, 2005) out of 100 subsets and their ranking. Sequence conservation is known to be important for nsSNV phenotype prediction. It is included with the PSSM and PSI-BLAST alignment results, as well as Jensen-Shannon divergence, which measures the level of conservation of a column in a multiple sequence alignment. We also include the emission probabilities for the wild-type and mutant amino acids at that position at the Pfam domain as positions which highly conserved in a domain are likely to show a large difference in the probability of observing a non-favourable amino acid.

The structural features used include relative solvent accessibility, which is known to be important in determining how likely an nsSNV is to be deleterious. We also include the secondary structure in which the nsSNV is found and its φ/ϕ backbone angles. These are included because certain amino acids are more restricted than others in terms of the areas of the Ramachandran plot they are able to occupy. For example, glycine is very flexible and can adopt conformations impossible for other amino acids to fit. Proline, on the other hand, has a much less flexible backbone, so mutation to a proline may lead to changes in backbone torsion angles. Because these two amino acids are the extreme cases, we also explicitly include as features whether or not the wild-type or mutant amino acid is glycine or proline.

| **Test Data** | **Features** | **Precision** | **Recall** | **MCC** | **Balanced Accuracy** |
| --- | --- | --- | --- | --- | --- |
| **Unseen Protein** | **All** | 0.80 | 0.72 | 0.64 | 0.81 |
| **Unseen SAV** | **All** | 0.81 | 0.74 | 0.66 | 0.82 |
| **Unseen SAV** | **Feature Selection** | 1.00 | 0.63 | 0.72 | 0.82 |
| **Unseen SAV** | **No Systems** | 0.79 | 0.68 | 0.60 | 0.79 |

**Table S2**

**Cross-validation on unseen proteins, unseen variants and with feature selection**

Because PPI centrality is a protein-specific feature, there may be over-training, with best performance on proteins present in the training set. To eliminate this possibility, two 10-fold cross-validations were carried out, one with no overlap between proteins in the training and test sets (Unseen Protein) and the other with overlap between proteins but not SAVs (Unseen SAV). Only a very slight drop in performance is seen for Unseen Protein compared to Unseen SAV, showing there is little over-training and the SVM is able to generalise to previously unseen proteins. The decreases in MCC (p=0.03, Wilcoxon test) and balanced accuracy (p=0.04, Wilcoxon test) are small but statistically significant.

In cross-validation with feature selection, stability selection was carried out using mRMR (see Methods) and the features selected in every subset used to train a model. This showed 100% precision in all cases, but much lower recall. Balanced accuracy is slightly lower than that for the full model (Wilcoxon test, p=0.02), but MCC (Matthews correlation coefficient) is significantly higher (Wilcoxon test, p<10^-4^).

To show the importance of the systems-level features, these were removed and cross-validation carried out again (No Systems). In all four measures, this gave significantly worse performance than the full version (Wilcoxon test, p<0.01 in all cases).

Mean values are shown across all 10 cross-validation sets.

|  | **Structure** | **Precision** | **Recall** | **MCC** | **Balanced Accuracy** |
| --- | --- | --- | --- | --- | --- |
| **Phyre2** | **+** | 0.809 | 0.800 | 0.649 | 0.824 |
| **Phyre2** | **-** | 0.803 | 0.797 | 0.641 | 0.820 |
| **PDB** | **+** | 0.825 | 0.900 | 0.550 | 0.785 |
| **PDB** | **-** | 0.816 | 0.907 | 0.539 | 0.750 |
| **Combined** | **+** | 0.812 | 0.818 | 0.647 | 0.824 |
| **Combined** | **-** | 0.806 | 0.817 | 0.639 | 0.820 |

**Table S3**

**Comparison of cross-validation performance with and without structural features**

Including structural features gives a small but statistically insignificant increase in performance in 10-fold cross-validation, with nsSNVs having structural information either from an experimentally solved structure (PDB) or a homology model (Phyre2). Values shown are mean across all 10 cross-validation sets.

|  | **SuSPect-All AUC** | **Specialised SVM AUC** | **DeLong’s test p-value** |
| --- | --- | --- | --- |
| **PDB** | 0.91 | 0.90 | 0.60 |
| **Phyre2** | 0.80 | 0.81 | 0.16 |
| **N/A** | 0.86 | 0.84 | 0.049 |

**Table S4**

**Performance of SuSPect-FS compared to methods trained solely on SAVs with structural information from PDB, Phyre2 or neither**

SVMs were trained only on those SAVs with a structure available in either the PDB or from Phyre2, and tested on the subset of VariBench with the relevant structure available. There was no improvement seen when SVMs were trained only on the subset of SAVs from PDB or Phyre2 (DeLong’s test, p=0.60 and p=0.16 respectively). In all three cases, SuSPect-All performs as well as or better than the specialised SVM, showing that specifically training a separate SVM for each case does not give improved performance.

Two further SVMs were trained on the PDB-only training data with either the NetSurfP predicted RSA or the NACCESS calculated RSAs removed. Inclusion of the NetSurfP RSA gave better performance than inclusion of the NACCESS RSAs (AUC=0.92, 0.90 respectively, DeLong’s test, p=3.3x10^-4^).

|  | **Deleterious** | **Neutral** | **SIFT AUC** | **SuSPect-No Networks AUC** | **SuSPect-FS No Networks AUC** |
| --- | --- | --- | --- | --- | --- |
| **HIV-1 protease** | 225 | 111 | 0.66 | 0.80 | 0.80 |
| **LacI** | 1774 | 2267 | 0.69 | 0.75 | 0.78 |
| **T4 lysozyme** | 638 | 1377 | 0.65 | 0.73 | 0.77 |
| **Combined** | 2637 | 3755 | 0.68 | 0.74 | 0.77 |

**Table S5**

**Performance of SuSPect on mutations in non-human proteins**

SuSPect-No Networks and SuSPect-FS No Networks both outperform SIFT at predicting functional impact of 6392 nsSNVs in three non-human proteins (DeLong’s test, p<10^-9^ for SuSPect-No Networks, p<2.2x10^-16^ for SuSPect-FS No Networks).

As SuSPect was trained to identify disease-causing variants in humans, it is unsurprising that performance is worse for these three non-human proteins than on human proteins (DeLong’s test, p<10^-9^). These non-human variants were downloaded from <http://sift.bii.a-star.edu.sg/index.html> and classified as deleterious if they showed any impact on protein function. As a change in protein function in a human protein is not the same as being disease-causing, this is a very different problem from what SuSPect was trained to perform, but SuSPect still performs reasonably well on these variants.

**Examples of SAVs**

Some examples of SAVs predicted correctly by SuSPect and incorrectly by other methods are described here.

- The Lys1019Glu variant in Q8NFU7 is in the Neutral dataset of VariBench. SuSPect-FS scores it 30, whereas SIFT (0), PolyPhen-2 (0.901) and Condel (0.857) all predict it to be damaging. MutationAssessor also predicts this SAV to be neutral (0.895).
- The Ser146Asn SAV in Q96RU7 is also in the Neutral dataset and is correctly predicted as such by SuSPect-FS (14) and MutationAssessor (1.375), whereas SIFT (0), PolyPhen-2 (0.997) and Condel (0.966) all incorrectly predict it to be damaging.
- In P02452, the Gly638Ser and Gly1100Ser are both in the VariBench pathogenic dataset. These SAVs are incorrectly annotated as neutral by PolyPhen-2 (0, 0) and Condel (0.004, 0), but are correctly predicted by SuSPect-FS (80, 86), SIFT (0.01, 0) and MutationAssessor (4.535, 4.105).
- The Gly452Ser variant in P10275 is incorrectly predicted to be neutral by SIFT (0.72), PolyPhen-2 (0), MutationAssessor (0) and Condel (0.001). SuSPect-FS, however, predicts it to be damaging (60).

**Figure S1**

**Distribution of scores from SuSPect-No Networks**.

This method is used on the SuSPect web-server for non-human proteins, which lack network information.
